# 

**Published:** 1832-01-01

**Authors:** 


### CONTENTS

OF THE

### MEDICO-CHIRURGICAL REVIEW.

No. XXXI. JANUARY 1, 1832.

### REVIEWS.

I.

Dr. Bright's Report's of Medical Cases, illustrating the Symptoms and Cure of Diseases (*second Article*) .. .. .. .. .. .. .. .. .. .. .. .. 1

II.

The Book of Analysis; or a New Method of Experience, &c. &c. By Dr. Todd .. 36

III.

Baron Larrey's Chirurgical Report of the Events of July 1830, in the Military Hospital of Gros Caillou .. .. .. .. .. .. .. .. .. .. .. .. .. 49

IV.

Dr. Paget on Congenital Malformations of the Heart .. .. .. .. .. .. .. 68

V.

Mr. Robertson on the Health of English Manufacturers .. .. .. .. .. .. .. 77

VI.

Dr. Walter Vaughan on Head-aches and their Cure .. .. .. .. .. .. .. 83

VII.

M. Andral on Hyperemia .. .. .. .. .. .. .. .. .. .. .. .. .. .. 87

VIII.

Report of the Select Committee of the House of Representatives, relative to the Legalizing of the Study of Anatomy .. .. .. .. .. .. .. .. .. .. 101

IX.

Essays on the Effects of Iodine in Scrofulous Diseases, &c. By M. Lugol. Translated by Dr. O'Shaughnessey .. .. .. .. .. .. .. .. .. .. .. .. .. 109

X.

Dr. Marshall on the Cholera in Glasgow .. .. .. .. .. .. .. .. .. .. 130

XI.

Principles of Lithotrity. By Baron Heurteloup .. .. .. .. .. .. .. 134

XII.

Dr. Morton on protracted Lactation, &c. .. .. .. .. .. .. .. .. .. .. 154

XIII.

Dr. Collier's Celsus .. .. .. .. .. .. .. .. .. .. .. .. .. .. .. 156

XIV.

EPIDEMIC CHOLERA.

Preliminary Observations .. .. .. .. .. .. .. .. .. .. .. .. .. .. 163

1. Professor Lichtenstadt—Epidemic at Orenburg .. .. .. .. .. .. .. 164

2. Sir WILLIAM CRICHTON—Cholera in Russia .. .. .. .. .. .. .. .. 168
3. Report of Dr. ALBERS, the Prussian Commissioner—Cholera in Moscow .. .. 170
4. Report of the Russian Commissioners at Moscow .. .. .. .. .. .. .. 172
5. Report of Dr. WALKER .. .. .. .. .. .. .. .. .. .. .. .. .. 175
6. Sir WILLIAM PYM's Letter to Government .. .. .. .. .. .. .. .. .. 177
Opinions of Sir Henry Halford and the College of Physicians .. .. .. .. 178

ANALYTICAL REVIEWS OF THE MORE IMPORTANT WORKS ON CHOLERA.

I. Mr. KENNEDY's History of the Contagious Cholera .. .. .. .. .. .. 180
II. Dr. NEALE on the Linnæan Doctrine of Animate Contagions.. .. .. .. 181
III. Mr. BELL on Cholera Asphyxia, or Epidemic Cholea .. .. .. .. .. 186
Pathology .. .. .. .. .. .. .. .. .. .. .. .. .. .. 188
Mode of Propagation.. .. .. .. .. .. .. .. .. .. .. .. 189
Treatment.. .. .. .. .. .. .. .. .. .. .. .. .. .. 192
IV. Mr. ORTON on the Epidemic Cholera of India .. .. .. .. .. .. .. 193
Symptomatology .. .. .. .. .. .. .. .. .. .. .. .. .. 194
Pathology .. .. .. .. .. .. .. .. .. .. .. .. .. .. 197
Connexion of Cholera with other Diseases .. .. .. .. .. .. .. 198
Contagion .. .. .. .. .. .. .. .. .. .. .. .. .. .. 199
Atmospheric Influence .. .. .. .. .. .. .. .. .. .. .. 202
Influence of Locality.. .. .. .. .. .. .. .. .. .. .. .. 205
Predisposition .. .. .. .. .. .. .. .. .. .. .. .. .. 208
Treatment .. .. .. .. .. .. .. .. .. .. .. .. .. .. 209
V. Mr. SEARLE on Cholera.
Causes of Cholera .. .. .. .. .. .. .. .. .. .. .. .. .. 211
Indications of Treatment.. .. .. .. .. .. .. .. .. .. .. 212
VI. Dr. LEFEVRE.
The St. Petersburg Choltra .. .. .. .. .. .. .. .. .. .. .. 213
VII. Dr. YOUNG on Cholera .. .. .. .. .. .. .. .. .. .. .. .. 217
VIII. Miscellaneous Works on Cholera—
Dr. KENNEDY's Notes .. .. .. .. .. .. .. .. .. .. .. .. 221
Dr. ALLEN .. .. .. .. .. .. .. .. .. .. .. .. .. .. .. 221
Baron LARREY .. .. .. .. .. .. .. .. .. .. .. .. .. .. 221
Dr. GRANVILLE .. .. .. .. .. .. .. .. .. .. .. .. .. .. 222
Dr. FERGUSSON .. .. .. .. .. .. .. .. .. .. .. .. .. .. 222
Dr. FORSTER .. .. .. .. .. .. .. .. .. .. .. .. .. .. 223
Mr. PETTIGREW.. .. .. .. .. .. .. .. .. .. .. .. .. .. 223
Mr. BELL's Letter to Sir Henry Halford .. .. .. .. .. .. .. .. 224
*(Continued in Periscope, p. 266.)*

### PERISCOPE.

1. On Phlegmonous Tumours in the right Iliac Fossa: by Mr. Ferrall.. .. .. .. 225
2. On Dothinenterite: by Dr. Corrigan .. .. .. .. .. .. .. .. .. .. .. 227
3. Mollescence of the Brain—Serous Apoplexy .. .. .. .. .. .. .. .. .. 228
4. Cancer of the Stomach mistaken for Hypertrophy of the Heart .. .. .. .. 231
5. Observations on Syncope and Cerebral Congestion: by M. Piorry .. .. .. 233
6. Dr. Davis's Principles and Practice of Obstetric Medicine.. .. .. .. .. .. 235
7. M. Piorry on Hemicrania.. .. .. .. .. .. .. .. .. .. .. .. .. .. 232
8. Mr. Bransby Cooper's Lectures on Anatomy.. .. .. .. .. .. .. .. .. 239
9. Maingault's Illustrations of the different Amputations, represented by Plates, 242

10. Mr. Liston's Elements of Surgery .. .. .. .. .. .. .. .. .. .. 242
11. Mr. Beaumont on a new Mode of treating Fractures .. .. .. .. .. .. 247
12. Case of Calculus formed on a Needle swallowed .. .. .. .. .. .. .. 251
13. M. Delpech on Varicocele, with Cases .. .. .. .. .. .. .. .. .. 252
14. Encysted Abscess of the Brain.. .. .. .. .. .. .. .. .. .. .. .. 255
15. Ulcer of the Rectum cured by Sarsaparilla and the Bougie.. .. .. .. .. 256
16. Microscopical Researches on the Blood in Disease .. .. .. .. .. .. .. 256
17. Dr. Perry's Clinical Lectures on Burns and Scalds .. .. .. .. .. .. 257
18. Dr. Hope's Treatise on Diseases of the Heart .. .. .. .. .. .. .. .. 262

19. Appendix to the Article on Cholera *(continued from page 224)*.

Cholera in England .. .. .. .. .. .. .. .. .. .. .. .. .. .. 266

Sunderland Epidemic .. .. .. .. .. .. .. .. .. .. .. .. .. 267

Loimometer; or Instrument for measuring the Degree of Contagion .. .. 267

State of the Loimometer 20th of October, 1831 .. .. .. .. .. .. .. .. 267

Ditto ditto 14th of November, 1831 .. .. .. .. .. .. .. 267

Contingent Contagion .. .. .. .. .. .. .. .. .. .. .. .. .. 268

On the discrimination of Cholera by Symptoms .. .. .. .. .. .. .. .. 269

Importation of Cholera from the Continent—the King unable to decide.. .. 269

" Travelling Cholera," a new Discovery .. .. .. .. .. .. .. .. .. 269

Prognostications of 1831 .. .. .. .. .. .. .. .. .. .. .. .. .. 270

The King's Speech respecting Cholera in England .. .. .. .. .. .. .. 270

Medical Constitution of the Year 1831 in England .. .. .. .. .. .. .. 270

Cases of Asiatic Cholera, by Drs. Marshall, Brown, &c. &c. .. .. .. .. 271

Extreme Cases of Cholera compared with extreme Cases of other Diseases—

as of Variola .. .. .. .. .. .. .. .. .. .. .. .. .. .. 271

Case of Hollis, the Tailor, in Poland Street .. .. .. .. .. .. .. .. .. 271

Case of a Medical Gentleman in Piccadilly, who died with the Symptoms of

Asiatic Cholera in August last.. .. .. .. .. .. .. .. .. .. .. .. 272

Case of Asiatic Cholera witnessed by Mr. Pretty.. .. .. .. .. .. .. .. 272

Dr. Marshall's first Case, presenting all the Symptoms of the Blue Cholera.. 272

Statements of Dr. Brown and Dr. Barry respecting the Disease in Sunderland, 272

Remarks on the Identity of Ancient and Modern Cholera.. .. .. .. .. .. 273

Relative Mortality in India, Europe, and England .. .. .. .. .. .. .. 274

Dr. Johnson's Propositions submitted to the Westminster Society .. .. .. 275

Propositions of the Central Board of Health.. .. .. .. .. .. .. .. .. 277

Parallel of the two Series of Propositions .. .. .. .. .. .. .. .. .. 279

Picture of Cholera in Sunderland, by Dr. Tinn .. .. .. .. .. .. .. .. 280

Ditto ditto ditto by the Times' Reporter.. .. .. .. .. 281

Ditto ditto ditto by Dr. O'Shaughnessey.. .. .. .. .. 281

Reflections on the foregoing Pictures .. .. .. .. .. .. .. .. .. .. 281

How is Cholera propagated? by an American Physician .. .. .. .. .. .. 281

Philosophy of the Epidemic .. .. .. .. .. .. .. .. .. .. .. .. 283

Bibliographical Record.. .. .. .. .. .. .. .. .. .. .. .. .. .. 284

### EXTRA-LIMITES.

I. Dr. Hacket's Animadversions on Dr. Stevens .. .. .. .. .. .. .. .. 289
Mr. Greatrex's Letter to Dr. Stevens.. .. .. .. .. .. .. .. .. .. 295
II. Dr. Sanders' Letter to Sir H. Halford, Bart. on Cholera .. .. .. .. .. 297

III. Dr. Brown's Letter to Drs. Johnson & Tweedie, on the Sunderland Epidemic 301
VI. Torti on Pernicious Fevers: by Dr. Negri .. .. .. .. .. .. .. .. .. 303
Editorial Observations on the reigning Epidemic .. .. .. .. .. .. .. 304
V. Extracts from Medical Reports on the Cholera Morbus which prevailed at Danzig from May to September, 1831. By Dr. Hamett .. .. .. .. .. 305

